# Radiation Dose Reduction in CT Torsion Measurement of the Lower Limb: Introduction of a New Ultra-Low Dose Protocol

**DOI:** 10.3390/diagnostics11071209

**Published:** 2021-07-03

**Authors:** Gabriel Keller, Simon Götz, Mareen Sarah Kraus, Leonard Grünwald, Fabian Springer, Saif Afat

**Affiliations:** 1 Department of Diagnostic and Interventional Radiology, University Hospital Tübingen, Eberhard Karls University Tübingen, 72076 Tübingen, Germany; simon.goetz@student.uni-tuebingen.de (S.G.); mareen.kraus@med.uni-tuebingen.de (M.S.K.); saif.afat@med.uni-tuebingen.de (S.A.); 2 Department of Traumatology and Reconstructive Surgery, BG Trauma Center Tübingen, Eberhard Karls University Tübingen, 72076 Tübingen, Germany; lgruenwald@bgu-tuebingen.de; 3 Department of Diagnostic Radiology, BG Trauma Center Tübingen, Eberhard Karls University Tübingen, 72076 Tübingen, Germany

**Keywords:** radiation exposure, radiation dosage, rotation, torsion, lower limb, lower extremity, ultra-low dose, ULD

## Abstract

This study analyzed the radiation exposure of a new ultra-low dose (ULD) protocol compared to a high-quality (HQ) protocol for CT-torsion measurement of the lower limb. The analyzed patients (*n* = 60) were examined in the period March to October 2019. In total, 30 consecutive patients were examined with the HQ and 30 consecutive patients with the new ULD protocol comprising automatic tube voltage selection, automatic exposure control, and iterative image reconstruction algorithms. Radiation dose parameters as well as the contrast-to-noise ratio (CNR) and diagnostic confidence (DC; rated by two radiologists) were analyzed and potential predictor variables, such as body mass index and body volume, were assessed. The new ULD protocol resulted in significantly lower radiation dose parameters, with a reduction of the median total dose equivalent to 0.17 mSv in the ULD protocol compared to 4.37 mSv in the HQ protocol (*p* < 0.001). Both groups showed no significant differences in regard to other parameters (*p* = 0.344–0.923). CNR was 12.2% lower using the new ULD protocol (*p* = 0.033). DC was rated best by both readers in every HQ CT and in every ULD CT. The new ULD protocol for CT-torsion measurement of the lower limb resulted in a 96% decrease of radiation exposure down to the level of a single pelvic radiograph while maintaining good image quality.

## 1. Introduction

Torsional malalignment is a common lower limb abnormality in children and adults. Causes of torsional malalignment are multifactorial and may originate from overuse, trauma, muscular imbalance, or congenital disorders [1,2,3,4,5]. It is known that torsional malalignment is a risk factor for the development of osteoarthritis and related disorders [6,7,8,9,10,11]. A surgical correction may therefore be necessary to slow down or avoid joint deterioration in the long run. Hence, measurement of lower limb torsion is recommended as a crucial part of the clinical workup, e.g., after recurrent patellar dislocations [12,13,14]. Up to date, diagnosis of torsional malalignment is usually based on both clinical and radiological data.

Computed tomography (CT) is considered the gold standard for routine clinical work-up and assessment of lower limb malalignments [15,16,17]. CT is widely available, quick, and cost-effective compared to other methods, such as magnetic resonance imaging (MRI). To perform torsion measurements of the lower limbs, the CT scan must include the hip, the knee, and the ankle regions, which results in a considerable exposure to ionizing radiation that is known to increase the risk of radiation-induced carcinogenesis [18,19]. Thus, it is inevitable to reduce radiation exposure as low as reasonably achievable (ALARA principle) while maintaining diagnostic accuracy. In the last decade, various technical approaches have been developed and implemented to reduce radiation dose in CT imaging, including modulation of tube current, automatic adjustment of tube voltage, reduction of *z*-axis scan coverage as well as overranging, and modern post-processing technologies, such as iterative reconstruction [20,21]. Recently, an ultra-low dose CT (ULD CT) simulation study has shown in an intra-individual setting that radiation dose reduction down to 1% of the original radiation dose and the non-inferiority regarding the diagnostic accuracy of torsion measurements of the lower limb [22]. Based on this simulated CT acquisition protocol, we implemented a ULD CT protocol in our clinical routine at a level-1 trauma center with a special focus on automatic adjustment of tube voltage, tube current modulation, and iterative image reconstruction. Furthermore, scan range was reduced to the absolute minimum based on anatomical landmarks.

After this simulation approach, the aim of the present study was to evaluate the real radiation exposure, diagnostic confidence (DC), and contrast-to-noise ratio (CNR) of this new clinically implemented ULD CT protocol and to compare the results to our previous standard high-quality CT (HQ CT) protocol for torsion measurements of the lower limb.

## 2. Materials and Methods

### 2.1. Study Population

In total, 60 consecutive patients with clinically indicated CT-torsion measurements were retrospectively included. Here, 30 consecutive patients before (HQ CT study protocol) and 30 consecutive patients after introduction of the new ULD CT protocol in June 2019 were evaluated (recruiting period March–October 2019). Exclusion criteria were metal implants in the scanning field. Demographic parameters were retrospectively recorded: age at time of CT, gender, and self-reported body mass index (BMI) from patients’ medical history. Radiation dose parameters, such as computed tomography dose index (CTDIvol), dose length product (DLP), and dose equivalent, were taken from the automated dose report. Scan length (SL) was calculated for each region (hip, knee, ankle) from DLP and CTDIvol. The study protocol was approved by the institutional review board (review board application number 930/2019BO2), and for this retrospective analysis of clinically acquired, data the need for written informed consent was waived.

### 2.2. Estimation of the Effective Radiation Dose

The effective radiation dose was estimated using the software package Radimetrics (Radimetrics Enterprise Platform, Bayer Pharma, Leverkusen, Germany). Effective radiation dose was calculated for every CT independent of the study protocol and separately for each region (hip, knee, ankle). The total effective dose is defined as the sum of radiation doses of all organs in the scan range. All organ doses were weighted by tissue weighting factors from the International Commission on Radiological Protection (ICRP103) and were directly calculated using Monte Carlo simulations.

### 2.3. Technical Parameters of the HQ Protocol for CT Torsion Measurement of the Lower Limb

CT image acquisition was performed using a 128-slice, single source CT (SOMATOM Definition Edge, Siemens Healthineers, Forchheim, Germany) using an HQ protocol with an automated tube current modulation for individual patient size and shape (CARE Dose4D, Siemens Healthineers, Forchheim, Germany) in the hip region. Tube voltage was set to 120 kV with fixed tube currents for the knee (95 mAs) and ankle (95 mAs). The reference tube current was set for the hip region at 220 mAs. Pitch was 1.0, rotation time 0.5 s, and collimation 128 × 0.6 mm. No iterative image reconstruction was used. Slice thickness was 3 mm, images were displayed in bone window (center/width: 450 HU/1500 HU).

### 2.4. Technical Parameters of the New ULD Protocol for CT Torsion Measurement of the Lower Limb

**Figure 1:**
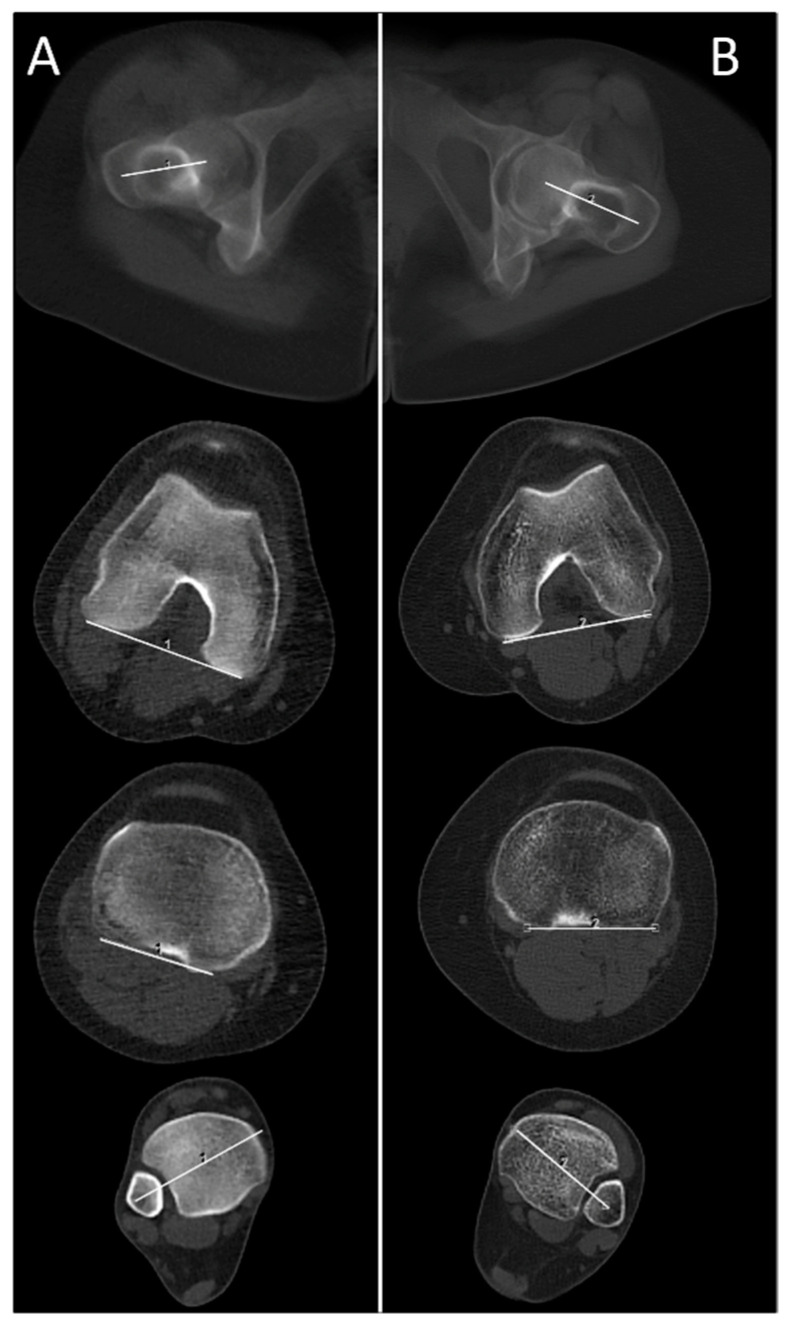
Comparison of a patient (**A**); female, 17 years, BMI = 25.2 kg/m^2^, no metal implants, total dose equivalent = 0.19 mSv examined with the newly introduced ultra-low dose (ULD) study protocol and a patient (**B**); female, 37 years, BMI 23.8 kg/m^2^, no metal implants, total dose equivalent = 4.09 mSv examined with the previous standard high-quality (HQ) study protocol for CT-torsion measurement (BMI = body mass index).

The new ULD protocols were acquired using the identical CT scanner (SOMATOM Definition Edge, Siemens Healthineers, Forchheim, Germany) with a pitch of 1.0, rotation time of 0.5 s, collimation of 128 × 0.6 mm, and a scan time of 2.21 s (hip), 2.11 s (knee), and 1.77 s (ankle). An automated tube current modulation (CARE Dose4D, Siemens Healthineers, Forchheim, Germany) was used for all regions (hip, knee, ankle). Furthermore, an automated tube voltage selection (CARE kV, Siemens Healthineers, Forchheim, Germany) was additionally used for all regions and was set to optimize the tube current for the depiction of osseous structures. Reference settings were set as following: hip (100 kV, 20 mAs), knee (80 kV, 20 mAs) and ankle (80 kV, 10 mAs). Furthermore, raw-data-based iterative image reconstruction (SAFIRE—Sinogram Affirmed Iterative Reconstruction, Siemens Healthineers, Forchheim, Germany) was used at strength 3 for all regions. Image reconstruction was performed using a medium sharp kernel, a 3 mm slice thickness, and a bone window (center/width: 450 HU/1500 HU) (see Figure 1).

**Figure 2:**
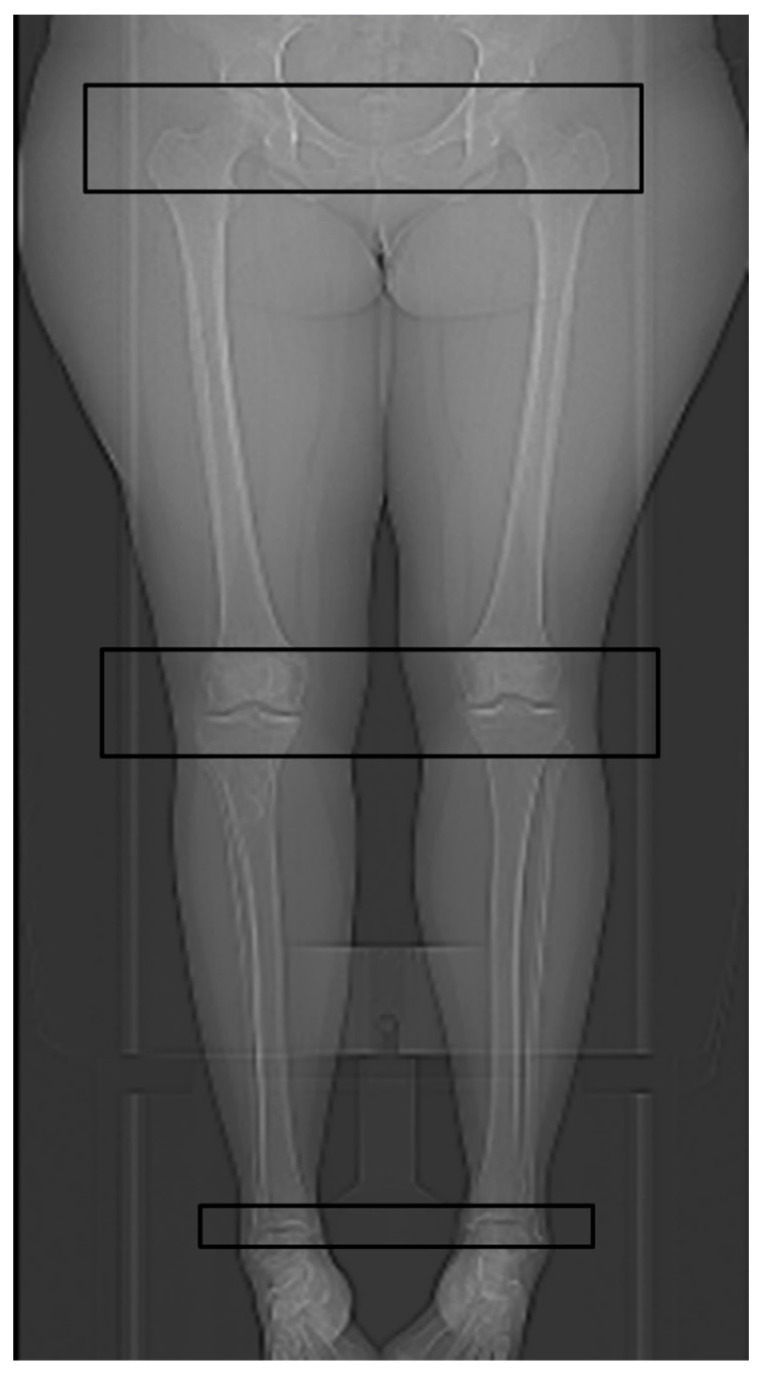
Anterior-posterior (AP) scout view with the marked scan length for the hip region, the knee region, and the ankle region according to the standard operating procedure (SOP) for the newly introduced ULD protocol for CT-torsion measurement of the lower limb.

In addition, the radiographers followed a new standard operating procedure (SOP) with a special focus on the absolute minimum necessary SL for the complete delineation of the relevant anatomical structures, based on prominent anatomical landmarks in the anterior-posterior (AP) scout view: At the hip, top of the femoral head to the upper margin of the lesser trochanter; at the knee, top of the patella to the middle of the fibular head; and at the ankle, 2 cm above the tibial plafond to the tip of the medial ankle (see Figure 2).

### 2.5. Measurement of Body Volume Parameters, CNR and DC

**Figure 3:**
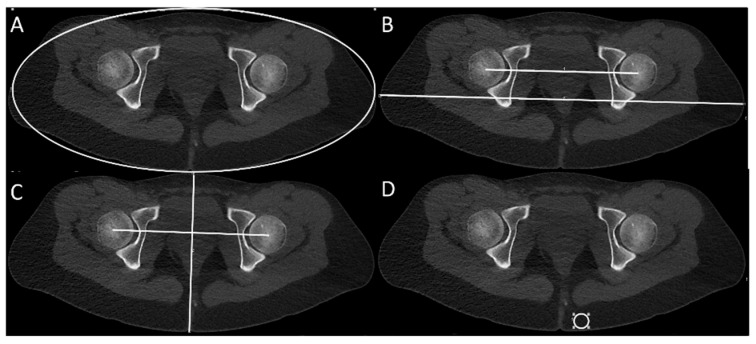
Measurement of body contour parameters at the femoral head level: best ellipsoid adaptation to the body contour (**A**), maximum body diameter right-left (RL) (**B**), and maximum body diameter anterior-posterior (**C**). Measurement of image noise in subcutaneous fatty tissue and contrast (cortical bone and surrounding skeletal muscle) at standardized anatomical positions for the calculation of CNR (contrast-to-noise ratio) (**D**).

All patients were examined feet first in supine position with the feet secured together. Using this standardized patient positioning, body volume was assessed at the level of the middle of the femoral heads. Body area was measured by the best ellipsoid adaptation to the body contour. Besides, the maximum right-left body diameter parallel to a line connecting the femoral head centers and the maximum anterior-posterior body diameter orthogonal to the right-left diameter were scaled (see Figure 3). To level out skeletal differences, the parameters body area, maximum right-left, and maximum anterior-posterior body diameter were divided by the distance between the femoral head centers.

For the assessment of CNR, the image noise was measured as the standard deviation of the Hounsfield units (HUs) within a 2 cm^2^ circular region of interest (ROI-) tool at a standardized anatomical position in the subcutaneous fatty tissue next to the anal cleft on the hip scan (see Figure 3). The image contrast was calculated by the difference of the average density of cortical bone and the average density of surrounding skeletal muscle at standardized anatomical positions in the posterior acetabulum and the gluteus maximus muscle (see Figure 3). CNR was then calculated by the quotient of contrast and image noise. Additionally, two readers with four and six years of experience in musculoskeletal imaging were blinded to the study protocol and rated their DC level regarding the identification of the relevant cortical bone in every CT-torsion measurement on a 5-point Likert scale from “very low” (1) to “very high” (5).

### 2.6. Statistical Analysis

Statistical analysis was performed using the software package JMP (Version 14.2.0, SAS Institute, Cary, NC, USA). The Shapiro-Wilk-W-test was performed for continuous variables to check for normality. Normally distributed variables are reported as arithmetic mean and standard deviation and were analyzed using a Student-t-test. Non-normally distributed variables are reported as a median with a range and were analyzed using the Wilcoxon-test. Correlations between continuous variables were analyzed by linear and polynomial fit of the second and third degree. Stated is the significant correlation with the lowest equational degree, respective of the lowest *p*-value, if there were no significant correlations. *p*-values < 0.05 indicate statistical significance.

## 3. Results

The groups of patients examined with the HQ protocol and with the new ULD protocol showed no significant difference in demographic parameters, such as age at time of CT, gender, and BMI. Self-reported BMI from the patient’s medical history could only be evaluated in 19/30 (HQ protocol) and 12/30 (ULD protocol) patients (as shown in Table 1).

**Figure 4:**
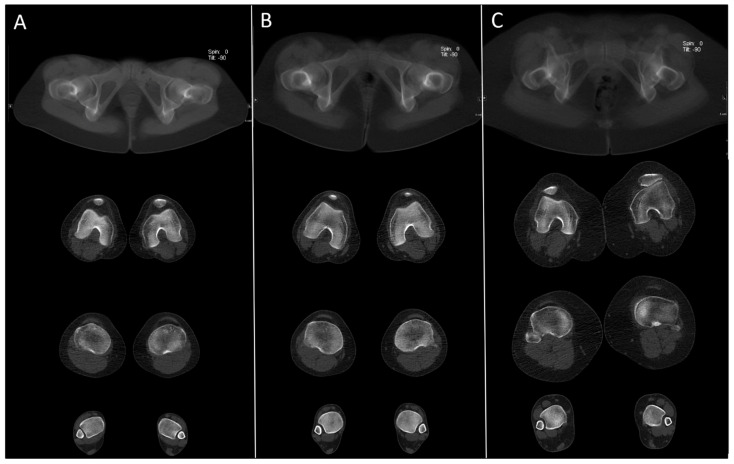
Example images of the newly introduced ULD protocol in three different patients with a BMI between 18.9 kg/m^2^ and 38.0 kg/m^2^ (**A**) female, 28 years, BMI = 18.9 kg/m^2^, CNR = 19.5, total dose equivalent = 0.27 mSv; (**B**) female, 17 years, BMI = 25.2 kg/m^2^, CNR = 16.5, total dose equivalent = 0.19 mSv; (**C**) female, 46 years, BMI 38.0 kg/m^2^, CNR = 15.9, total dose equivalent = 0.60 mSv).

However, body contour parameters, which could be measured in every CT, showed significant correlations to BMI and were not significantly different between the groups (see Table 2 and Figure 4). Mean CNR at the hip region was 12.2% lower in the new ULD protocol (see Table 3).

Both readers’ DC was “very high” (5 on the 5-point Likert scale) regarding the identification of the relevant cortical bone in both CT protocols (100.0%).

Actual acquisition settings for tube voltage and tube current were dependent on body size and are shown in Table 4.

Radiation dose parameters CTDIvol, DLP, and dose equivalent were significantly lower using the new ULD protocol if compared separately for hip, knee, and ankle regions as well as for the whole examination (see Table 3). Thereby, the median total dose equivalent in the new ULD protocol was only 3.9% of the HQ protocol.

With the implemented new SOP, the SL of all regions and total examination was significantly lower in the ULD protocol. The median total SL was reduced by about 6.5 cm to 84.8% of the SL of the HQ protocol. Thereby, the median SL reduction was lowest in the hip region (minus 1.4 cm to 90.6% compared to the HQ protocol), respectively, in the knee region 4.1 cm to 74.5% and in the ankle region 6.5 cm to 84.8% (see Table 5).

Therefore, the median reduction of dose equivalent solely caused by SL reduction was 0.36 mSv for the hip, 0.23 mSv for the knee, and <0.01 mSv for the ankle region, accounting for a total median dose equivalent reduction of about 0.59 mSv caused by SL reduction if the HQ protocol with the new SOP was used.

## 4. Discussion

In this study, we compared a new ULD CT protocol to a HQ CT protocol for torsion measurement of the lower limb regarding radiation exposure, CNR, and DC. Our results show that by applying the new ULD CT protocol, a significant dose reduction to 0.17 mSv can be achieved at an acceptable CNR level whilst maintaining DC.

We retrospectively evaluated both subjective DC as well as objective parameters, such as CNR. Although CNR was significantly lower in ULD CT data sets, the DC in identifying relevant cortical landmarks remained high (5/5 on the Likert scale). Similarly, a recent CT simulation study of 30 patients showed no significant difference in terms of diagnostic accuracy of the measured torsion of the lower limbs between the simulated ULD CT data and the original data sets in an intra-individual setting, even for extremely low radiation doses [22]. In this study, image noise was retrospectively added to CT raw data to estimate the effect of a reduced tube current, i.e., simulating low dose CT images down to extremely low dose levels. However, such simulated ULD CT and real ULD CT are not the same; the effect of an additionally reduced tube voltage (depending on body size) could not be investigated in this simulation study but was additionally used in the ULD CT protocol of the current study. The automated tube voltage selection used in the ULD CT protocol focused on the delineation of osseous structures to ensure comparable image quality independent of body volume.

Many studies have already shown that ULD CT is an alternative imaging modality to conventional radiographs for imaging of extremities in well-defined settings [23,24,25,26,27]. To date, no study has yet examined ULD CT approaches for torsion measurements of the lower limb. Alagic et al. demonstrated that ULD CT is a useful alternative to conventional radiography for imaging of the peripheral skeleton with a comparable mean effective radiation dose between ULD CT and digital radiography of acute wrist and ankle fractures [23]. Furthermore, Konda et al. demonstrated that limb fractures can be diagnosed on ULD CT images as accurately as on conventional CT images [25].

In our study, the observed radiation dose reduction of the new ULD CT protocol cannot be attributed to anthropomorphic differences between the two groups, as both groups were comparable in terms of BMI, demographic data, and body contour parameters. Another factor that influences the effective radiation dose is the scan length. By implementing a new SOP with clear instructions and visual presentation of the scan length of interest, the overall scan length could be significantly reduced in the new ULD CT protocol. Hence, the radiation dose reduction caused by SL reduction was calculated at 0.59 mSv. The main part of the observed median radiation dose reduction of 3.61 mSv was due to the new ULD CT protocol. Radiation dose for complete torsion measurement of the lower limb using the new ULD CT protocol accounted for only 0.17 mSv, which is less than a single standard anterior-posterior radiograph of the pelvis (calculated at www.xrayrisk.com, accessed on 29 May 2021). Thus, ULD CT protocols for measurement of lower limb torsion seem to be applicable even in pediatric and adolescent patient groups.

When using the new ULD protocol, trabecular osseous structures seem more blurred, and therefore, in selected cases, a separate CT scan of the knee, hip, or ankle in diagnostic quality may need to be acquired if clinically necessary. Using the previous standard HQ CT protocol, diagnostic image quality was achieved without the need for additional scans of a specific joint. However, the higher dosage resulted in an image quality much higher than needed for sole torsion measurement in the majority of cases.

A major strength of the new ULD CT protocol is that the protocol is widely applicable due to the broad availability of CT scanners and easy implementation of the new ULD CT protocol in almost every CT scanner generation (even if used without automatic tube voltage selection/automatic tube current modulation). Furthermore, our results emphasize the potential role of ULD CT as an appropriate alternative imaging approach for patients with torsional malalignments of the lower limb. This also applies to patients with contraindication to MRI or in circumstances where MRI or alternatives are not available, especially in the setting of a diagnostic work-up of recurrent patellar dislocations in adolescent or pediatric patients.

Further dose reduction and improvement of CNR may even be achieved by using modern CT scanners with a fully integrated circuit detector system, inherently generating less image noise, or by advanced post-processing algorithms (e.g., the advanced modeled iterative reconstruction technique (ADMIRE), Siemens Healthineers, Forchheim, Germany). The use of artificial intelligence and neural networks may also have potential to further improve CNR [28,29,30]. We believe this may be subject to future research on ULD CT protocols for torsion measurements of the limbs and other indications.

The major limitation of this study is its monocentric retrospective setting with the comparison of two different groups of patients and their limited sample size. However, we accounted for possible effects of anthropomorphic differences between the groups by comparing various body contour parameters and we are convinced that the two groups are large enough to draw valid conclusions that withstand scrutiny. The validation of the recommended ULD protocol is limited by the subjectively rated DC of the radiological readers. Besides this, it must be mentioned that our previous standard HQ CT protocol was of a comparatively high effective radiation dose; however, the reduction down to 0.17mSv using the ULD protocol without markedly compromising image quality is of clinical relevance. Nevertheless, a multicenter approach with a larger patient cohort and different CT scanners is required to validate the results and confirm the applicability of the newly described ULD protocol in a clinical setting.

## 5. Conclusions

In conclusion, after previously proven non-inferiority of simulated ULD-CT regarding diagnostic accuracy of the lower limb torsion [22], we showed now that the new ULD protocol with approximately 100 kV/20 mAs (hip) and 80 kV/10 mAs (knee and ankle) in CT torsion measurement of the lower limb results in an approximately 96% decrease in effective radiation exposure compared to our HQ CT protocol. Thus, CT torsion measurement of the lower limb can now be performed at an effective radiation dose of 0.17 mSv that is comparable to the effective radiation dose of a single anterior-posterior pelvic radiograph. Furthermore, DC was equivalent between protocols despite the slightly reduced CNR and blurred image character of the ULD CT protocol.

## Figures and Tables

**Table 1:** Characterization of the population examined with the previous standard HQ study protocol and with the new ULD protocol for CT-torsion measurement of the lower limb.

	HQ Protocol	ULD Protocol	*p*-Value
N	30	30	-
Age at time of CT (yrs.) #	40.4 ± 15.9	38.8 ±15.8	0.784
Gender (male/female)	17/13	16/14	0.795
BMI (kg/m^2^) #	29.1 ± 5.4	28.7 ± 6.0	0.777
Body area at femoral head level; (cm^2^) #	738.8 ± 130.2	723.6 ± 131.8	0.515
Ratio area/distance femoral heads #	4.1 ± 0.7	4.1 ± 0.7	0.819
Max. RL body diameter at femoral head level (mm) #	388.8 ± 36.5	393.6 ± 31.4	0.344
Ratio max. RL diameter/distance femoral heads #	2.2 ± 0.3	2.2 ± 0.2	0.762
max. AP body diameter at femoral head level (mm) #	219.4 ± 28.8	219.3 ± 30.6	0.923
Ratio max. AP diameter/distance femoral heads #	1.2 ± 0.2	1.2 ± 0.2	0.900

# = normally distributed, reported in arithmetic mean and standard deviation, analyzed using Wilcoxon-test.

**Table 2:** Correlation of the measured body contour parameters in CT at the femoral head level with the body mass index.

	BMI (*p*-Value; Equational Fit)	Correlation Coefficient (r^2^)
Body area	<0.001 (linear fit)	0.73
Ratio area/distance femoral heads	<0.001 (linear fit)	0.74
Max. RL body diameter	<0.001 (linear fit)	0.48
Ratio max. RL diameter/distance femoral heads	<0.001 (linear fit)	0.33
Max. AP body diameter	<0.001 (linear fit)	0.77
Ratio max. AP diameter/distance femoral heads	<0.001 (linear fit)	0.67

**Table 3:** Parameters of the radiation dosage and CNR in the HQ protocol and in the new ULD protocol for CT-torsion measurement.

	HQ Protocol	ULD Protocol	*p*-Value
CNR #	19.8 ± 3.6	17.6 ± 4.1	0.033
CTDIvol hip (mGy) *	17.1 (9.5–35.7)	0.8 (0.6–1.5)	<0.001
CTDIvol knee (mGy) *	6.2 (3.6–13.5)	0.2 (0.2–0.4)	<0.001
CTDIvol ankle (mGy) *	6.4 (3.8–6.5)	0.2 (0.2–0.2)	<0.001
DLP hip (mGycm) *	279.5 (125–596)	10 (7–20)	<0.001
DLP knee (mGycm) *	98.5 (48–219)	2.5 (2–5)	<0.001
DLP ankle (mGycm) *	78 (43–104)	2 (1–2)	<0.001
DLP total (mGycm) *	457 (251–851)	15 (11–26)	<0.001
mSv hip *	3.79 (1.44–11.7)	0.15 (0.08–0.69)	<0.001
mSv knee *	0.90 (0.03–2.76)	0.03 (<0.01–0.11)	<0.001
mSv ankle *	0.05 (0.01–0.07)	<0.01 (<0.01–<0.01)	<0.001
mSv total *	4.37 (2.09–13.15)	0.17 (0.08–0.80)	<0.001

CTDIvol = computed tomography dose index, DLP = dose length product, * = non normally distributed, reported in median and range, analyzed using Student-*t*-test, # = normally distributed, reported in arithmetic mean and standard deviation, analyzed using Wilcoxon-test.

**Table 4:** Actual acquisition settings of tube voltage and tube current of the HQ protocol and the new ULD protocol in CT-torsion measurement of the lower limb.

	Tube Voltage HQ Protocol	Tube Voltage ULD Protocol	Tube Current HQ Protocol	Tube Current ULD Protocol
Hip	120 kV	26 × 100 kV 4 × 120 kV	292.4 ± 110.6 mAs	100 kV: 20 (14–27) mAs 120 kV: 21 (19–24) mAs
Knee	120 kV	80 kV	95.0 mAs	9 (9–20) mAs
Ankle	120 kV	80 kV	95.0 mAs	10 (10–10) mAs

**Table 5:** Scan length of the HQ protocol and the new ULD protocol in CT-torsion measurement.

	HQ Protocol	ULD Protocol	*p*-Value
SL hip *	14.9 (12.8–24.6)	13.5 (10.8–17.0)	<0.001
SL knee *	16.1 (11.5–26.3)	12.0 (11.1–22.2)	0.019
SL ankle *	12.2 (9.4–16.3)	11.1 (5.6–11.1)	<0.001
SL total *	42.9 (35.5–57.4)	36.4 (27.5–44.6)	<0.001

SL = scan length, * = non normally distributed, reported in median and range, analyzed using Student-t-test.

## Data Availability

Data sharing not applicable.

## Abbreviations and Acronyms

ALARA	“as low as reasonably achievable-principle”
AP	anterior-posterior
BMI	body mass index
CNR	contrast-to-noise ratio
CTDIvol	computed tomography dose index
DC	subjectively rated diagnostic confidence
DLP	dose length product
HQ	high-quality
RL	right-left
ROI	region of interest
SL	scan length
SOP	standard operating procedure
ULD	ultra-low dose

## References

[1] Breugem S.J., van Ooij B., Haverkamp D., Sierevelt I.N., van Dijk C.N. (2014). No difference in anterior knee pain between a fixed and a mobile posterior stabilized total knee arthroplasty after 7.9 years. Knee Surg. Sports Traumatol. Arthrosc..

[2] Erkocak O.F., Altan E., Altintas M., Turkmen F., Aydin B.K., Bayar A. (2016). Lower extremity rotational deformities and patellofemoral alignment parameters in patients with anterior knee pain. Knee Surg. Sports Traumatol. Arthrosc..

[3] Karaman O., Ayhan E., Kesmezacar H., Seker A., Unlu M.C., Aydingoz O. (2014). Rotational malalignment after closed intramedullary nailing of femoral shaft fractures and its influence on daily life. Eur. J. Orthop. Surg. Traumatol..

[4] Petersen W., Ellermann A., Gosele-Koppenburg A., Best R., Rembitzki I.V., Bruggemann G.P., Liebau C. (2014). Patellofemoral pain syndrome. Knee Surg. Sports Traumatol. Arthrosc..

[5] Werner S. (2014). Anterior knee pain: An update of physical therapy. Knee Surg. Sports Traumatol. Arthrosc..

[6] Brouwer G.M., van Tol A.W., Bergink A.P., Belo J.N., Bernsen R.M., Reijman M., Pols H.A., Bierma-Zeinstra S.M. (2007). Association between valgus and varus alignment and the development and progression of radiographic osteoarthritis of the knee. Arthritis Rheum..

[7] Cooke D., Scudamore A., Li J., Wyss U., Bryant T., Costigan P. (1997). Axial lower-limb alignment: Comparison of knee geometry in normal volunteers and osteoarthritis patients. Osteoarthr. Cartil..

[8] Eckhoff D.G. (1994). Effect of limb malrotation on malalignment and osteoarthritis. Orthop. Clin. N. Am..

[9] Gugenheim J.J., Probe R.A., Brinker M.R. (2004). The effects of femoral shaft malrotation on lower extremity anatomy. J. Orthop. Trauma.

[10] Moussa M. (1994). Rotational malalignment and femoral torsion in osteoarthritic knees with patellofemoral joint involvement. A CT scan study. Clin. Orthop. Relat. Res..

[11] Sharma L., Song J., Felson D.T., Cahue S., Shamiyeh E., Dunlop D.D. (2001). The role of knee alignment in disease progression and functional decline in knee osteoarthritis. JAMA.

[12] Dickschas J., Harrer J., Reuter B., Schwitulla J., Strecker W. (2015). Torsional osteotomies of the femur. J. Orthop. Res..

[13] Fithian D.C., Paxton E.W., Stone M.L., Silva P., Davis D.K., Elias D.A., White L.M. (2004). Epidemiology and natural history of acute patellar dislocation. Am. J. Sports Med..

[14] Jagodzinski M., Niemeyer P., Zeichen J., Balcarek P. (2014). German Society for Trauma Surgery S1-Guideline: Patellar Dislocation.

[15] Grisch D., Dreher T. (2019). Torsion and torsional development of the lower extremities. Orthopade.

[16] Hernandez R.J., Tachdjian M.O., Poznanski A.K., Dias L.S. (1981). CT determination of femoral torsion. AJR Am. J. Roentgenol..

[17] Widjaja P.M., Ermers J.W., Sijbrandij S., Damsma H., Klinkhamer A.C. (1985). Technique of torsion measurement of the lower extremity using computed tomography. J. Comput. Assist. Tomogr..

[18] Berrington de Gonzalez A., Mahesh M., Kim K.P., Bhargavan M., Lewis R., Mettler F., Land C. (2009). Projected cancer risks from computed tomographic scans performed in the United States in 2007. Arch. Intern. Med..

[19] Brenner D.J. (2010). Slowing the increase in the population dose resulting from CT scans. Radiat. Res..

[20] Kalra M.K., Maher M.M., Toth T.L., Hamberg L.M., Blake M.A., Shepard J.A., Saini S. (2004). Strategies for CT radiation dose optimization. Radiology.

[21] Mettler J.A., Griffin L. (2016). Muscular endurance training and motor unit firing patterns during fatigue. Exp. Brain Res..

[22] Keller G., Afat S., Ahrend M.D., Springer F. (1007). Diagnostic accuracy of ultra-low-dose CT for torsion measurement of the lower limb. Eur. Radiol..

[23] Alagic Z., Bujila R., Enocson A., Srivastava S., Koskinen S.K. (2020). Ultra-low-dose CT for extremities in an acute setting: Initial experience with 203 subjects. Skeletal Radiol..

[24] Brink M., Steenbakkers A., Holla M., de Rooy J., Cornelisse S., Edwards M.J., Prokop M. (2019). Single-shot CT after wrist trauma: Impact on detection accuracy and treatment of fractures. Skeletal Radiol..

[25] Konda S.R., Goch A.M., Leucht P., Christiano A., Gyftopoulos S., Yoeli G., Egol K.A. (2016). The use of ultra-low-dose CT scans for the evaluation of limb fractures: Is the reduced effective dose using ct in orthopaedic injury (REDUCTION) protocol effective?. Bone Joint J..

[26] Mansfield C., Ali S., Komperda K., Zhao H., Rehman S. (2017). Optimizing Radiation Dose in Computed Tomography of Articular Fractures. J. Orthop. Trauma.

[27] Yi J.W., Park H.J., Lee S.Y., Rho M.H., Hong H.P., Choi Y.J., Kim M.S. (2017). Radiation dose reduction in multidetector CT in fracture evaluation. Br. J. Radiol..

[28] Akagi M., Nakamura Y., Higaki T., Narita K., Honda Y., Zhou J., Yu Z., Akino N., Awai K. (2019). Deep learning reconstruction improves image quality of abdominal ultra-high-resolution CT. Eur. Radiol..

[29] Hong J.H., Park E.A., Lee W., Ahn C., Kim J.H. (2020). Incremental Image Noise Reduction in Coronary CT Angiography Using a Deep Learning-Based Technique with Iterative Reconstruction. Korean J. Radiol..

[30] Wolterink J.M., Leiner T., Viergever M.A., Isgum I. (2017). Generative Adversarial Networks for Noise Reduction in Low-Dose CT. IEEE Trans. Med. Imaging.

